# Adenoid cystic carcinoma of the cardia: Report of a rare case and review of the Chinese literature

**DOI:** 10.3892/ol.2014.2153

**Published:** 2014-05-19

**Authors:** YIMING ZHOU, YIWEN ZANG, JIANBIN XIANG, FENG TANG, ZONGYOU CHEN

**Affiliations:** 1 Department of General Surgery, Huashan Hospital Affiliated to Fudan University, Shanghai 200040, P.R. China; 2 Department of Pathology, Huashan Hospital Affiliated to Fudan University, Shanghai 200040, P.R. China

**Keywords:** adenoid cystic carcinoma, cardia, esophagus

## Abstract

Adenoid cystic carcinoma (ACC) is a relatively common head and neck tumor, however, is rare in the digestive tracts. There have been <100 cases of esophageal ACC reported to date and no cases of gastric ACC. The present study reports the exceptional case of a 53-year-old male with a primary ACC of the cardia. The patient underwent a radical total gastrectomy with D2 lymphadenectomy and Roux-en-Y esophagojejunal reconstruction. Immunohistochemical analysis identified a case of primary ACC that exhibited a positive expression for cytokeratin, calponin, cluster of differentiation 117, p63 and smooth muscle actin, with typical cribriform foci. No signs of recurrence have been detected during the 30-month follow-up. Thus, a precise diagnosis of ACC is primarily based on the results of immunohistochemical analysis and radical resection is considered to be the best treatment option for ACC of the digestive tracts. The current study also reviewed 17 cases of ACC of the esophagus reported in China, with special reference to the criteria for histological diagnosis and therapeutic options. The prognosis of esophageal ACC is poor due to early metastasis, mainly relying on the resectability of the tumor

## Introduction

Adenoid cystic carcinoma (ACC) is an uncommon form of malignant neoplasm that arises most commonly in the major and minor salivary glands of the head and neck (1), but rare in the esophagus. Only ~60 cases of esophageal ACC have previously been reported, which only accounts for <1% of all primary esophageal carcinomas reviewed (2). Usually a radical resection is performed, if the tumor is resectable. A result of which is that the small biopsy tissue samples may not display the characteristic architectural patterns of the tumor, and therefore a precise pre-operation diagnosis of ACC of the esophagus is difficult to achieve. The prognosis of esophageal ACC is poor, with organ mesastasis occurring more frequently with ACC than in other carcinomas (3). However, to date, ACC originating from the cardia has not been reported. As the invasive site of ACC is close to the esophagus and cardia, and the biological and pathological characteristics are similar, the current study presents a rare case of cardial ACC and a review of 17 other cases of ACC of the esophagus that have been reported in China so as to provide more information regarding the clinic manifestations and operable treatments for ACC of the upper digestive tract. Patient provided written informed consent.

## Case report

### Patient history and examination

**Figure 1:**
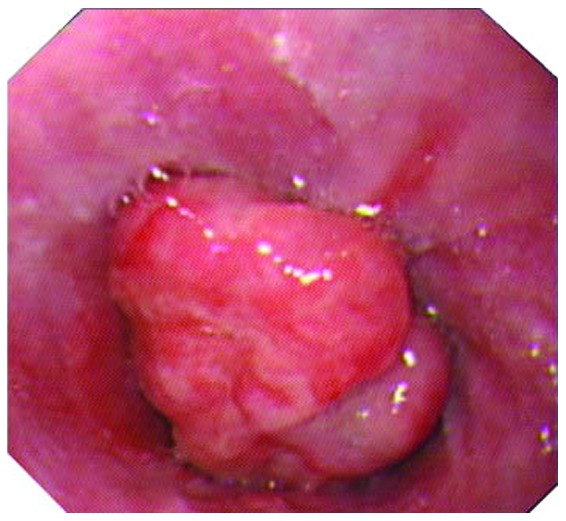
Endoscopy showing a protruded lesion with shallow depressions on the surface of the cardia.

**Figure 2:**
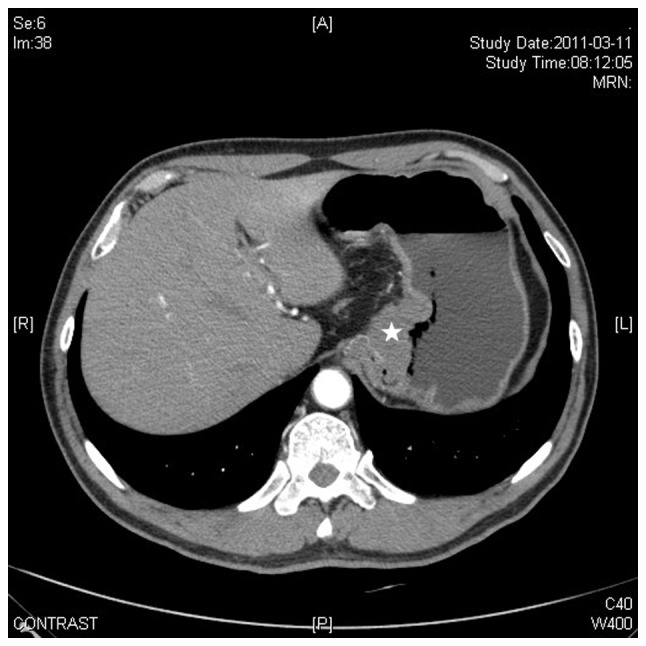
Enhanced computed tomography scan showing the abnormally thickened stomach wall of the cardia (indicated by the star symbol). The lymph nodes around the lesser curvature were enlarged.

A 53-year-old male was hospitalized at the Huashan Hospital Affiliated to Fudan University (Shanghai, China) with a four-month history of progressive dysphagia and proximal dull pain in the upper abdomen. The physical examination did not show any specific findings, and carcinoembryonic antigen, carbohydrate antigen 19-9 and markers for gastrointestinal carcinoma were normal. The findings from additional laboratory assessments were also normal. Endoscopy showed a protruded lesion with a shallow depression on the surface of the cardia, ~40–45 cm away from the patient’s incisors. The mucosa around the lesion was smooth, pink and gray-white, and the capillaries were diastolic (Fig. 1). The enhanced computed tomography (CT) scan showed that the stomach wall of the cardia was abnormally thickened and the lymph nodes around the lesser curvature were enlarged (Fig. 2). No apparent abnormalities were identified in the chest CT scan and abdominal B-mode ultrasound. A biopsy specimen indicated an adenocarcinoma with a partial structure of ACC. During the exploratory laparotomy, a 6×4-cm tumor was observed on the lesser curvature of the cardia, invading into the stomach body. A pale nodule, 5 mm in diameter, was identified in the left lateral lobe of the liver (potentially a metastatic nodule). No apparent signs of metastasis were found in the other organs in the abdomen. A local expanded resection of the liver nodule was performed to ascertain its pathological type and the frozen section results indicated that the nodule was an adenomatous hyperplasia. Thus, a radical total gastrectomy with a D2 lymphadenectomy and Roux-en-Y esophagojejunal anastomosis was performed.

### Treatment and follow-up

**Figure 3:**
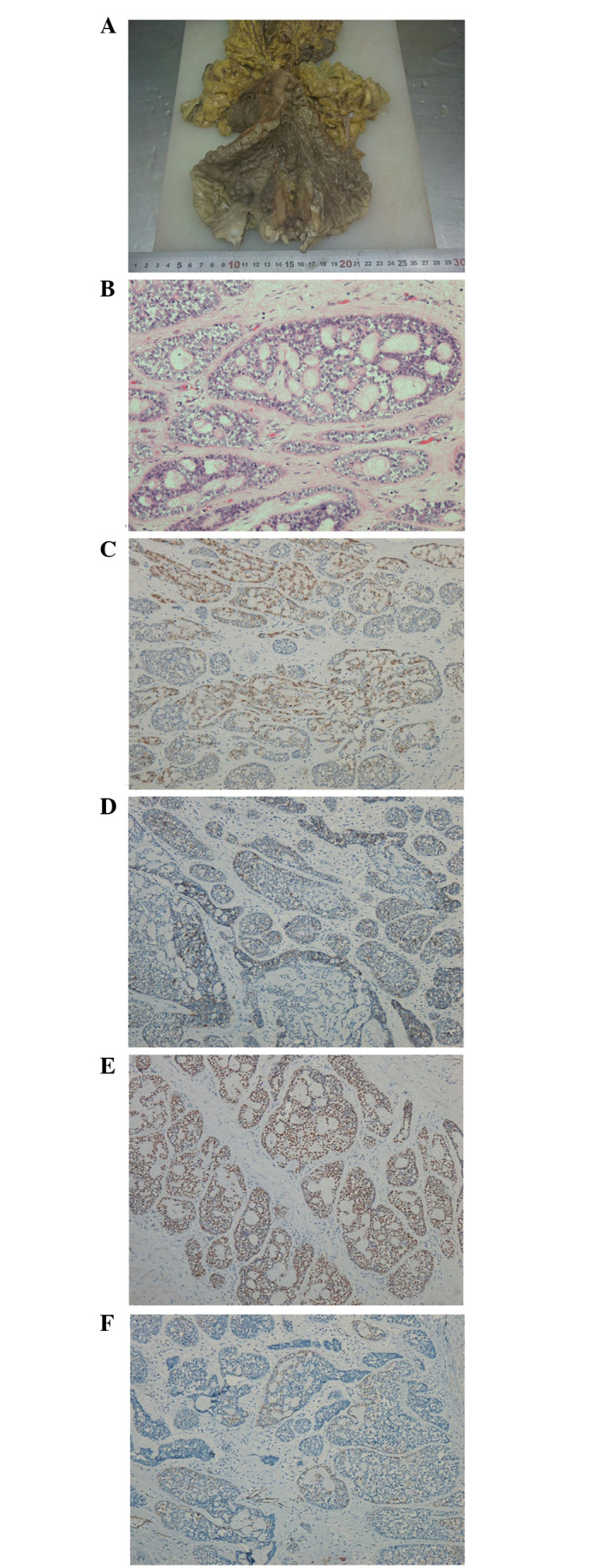
Immunohistological examinations. (A) Macroscopic tumor specimen obtained from surgical resction. (B) Micrograph of the resected specimen showing a cribriform pattern in a solid nest (hematoxylin and eosin staining; magnification, ×100). (C) Immunostaining for calponin (magnification, ×40); (D) cluster of differentiation 117 (magnification, ×40); (E) p63 (magnification, ×40); and (F) smooth muscle actin (magnification, ×40).

The resected tumor was a non-encapsulated fungoid-shaped mass, measuring 6×4 cm, located in the cardia and 2 cm from the upper incisal margin. The cut surface of the tumor had a hard, gray-white appearance (Fig. 3A). Microscopic examination of the lesion demonstrated an infiltrative malignant neoplasm invading serosa without metastases to the lymph nodes (pT4aN0M0, stage IIB according to the tumor-node-metastasis; TNM staging system). Histologically, the tumor primarily exhibited a cribriform pattern and partial tubular and solid patterns (Fig. 3B). The cells formed cylidromatous and tubule microscopic spaces, the lumen of which contained eosinophilic or basophilic AB-positive hyalinized materials. Specific lumens contained eosinophilic, periodic acid-Schiff (PAS)-positive materials. The inner layer of the tumor tissue was composed of a glandular epithelium, a layer of cubic or columnar cells forming glandular tubes, with abundant cytoplasm and enlarged hyperchromatic nuclei. By contrast, the external layer was composed of basaloid myoepithelium, exhibiting a uniformly small size, hyperchromatic, ovoid or spindle-shaped and scant cytoplasm. Results of immunohistochemistry (IHC) that was performed on the resected tumor are listed in Table I and demonstrated in Fig. 3C–F. These findings were consistent with an ACC developing from the cardia. The patient was discharged from hospital seven days following surgery and no signs of recurrence have been detected during the 30 months of follow-up.

## Discussion

Cancers of the adenoid cystic type commonly originate in the major salivary glands and account for 10–25% of all salivary gland tumors. These are indolent, locally aggressive tumors that only metastasize in the advanced cases. By contrast, ACC rarely originates from the esophagus (3). Since the first study, reported in 1954 by Gregg and Stamler (4), there have only been 17 cases reported in China and ~60 cases in other countries. Petursson (5) reviewed 45 cases of esophageal ACC. The mean age of patients with esophageal ACC was 65 years, and the male to female ratio was 3.4:1. Progressive dysphagia is the most common presenting symptom of ACC, which is comparable to esophageal squamous cell carcinoma (SCC); certain patients may also complain of upper abdominal discomfort. Superficial carcinoma in the early stage may be asymptomatic and identified during a routine gastroscopy examination. ACC originates most commonly in the middle third of the esophagus, less often in the lower third and rarely in the upper third. The present case was a primary ACC of the gastric cardia, involving the stomach body, which, to the best of our knowledge, has not previously been reported. Myoepithelial cells are rare in gastric glands. It was believed in the present case that the tumor originated from the lower esophagus and infiltrated into the cardia and the body of the stomach. As a result, the following is a further discussion about esophageal ACC.

The clinical data of the present case and 17 other cases of ACC of the esophagus documented in the literature are listed in Table II (6–18). The patients ranged in age from 42 to 68 years with an average age of 54.5 years. The gender ratio was 14 males to four females (3.5:1). The middle third of the esophagus was the most commonly affected region (10/18). The tumor appearance was protruded in nine cases and ulcerative in three of the 12 cases, where the macroscopic appearance was reported. These epidemiological results are consistent with a study by Morisaki *et al* (3). A total of 12 cases adopted barium radiography for preoperative diagnosis, seven cases of which indicated an esophageal malignant tumor, three indicated a benign tumor and one indicated cricopharyngeal achalasia. Although barium radiography may provide a precise direction for the position of the lesion, the topical diagnosis of esophageal ACC relies on microscopic examination. In total, 11 cases reported the performance of endoscopic biopsies for diagnosis, however, the diagnostic results were poor. Only three of the 12 reported biopsies indicated ACC, four indicated SCC, one indicated adenocarcinoma, one indicated leiomyoma, one indicated focal mild hyperplasia and the remaining biopsy was negative for any abnormality. The biggest challenge in diagnosing esophageal ACC from a biopsy specimen is associated with the small tissue samples, which may not exhibit the primary characteristic structural pattern of the tumor.

ACC is predominantly composed of glandular tube and myoepithelial cells. Polymorphism is a key characteristic of the tumor cells as they usually present in a cribriform, tubule or solid pattern. The cyst lumen that is formed by tumor cells often exhibits Alcian blue-positive or PAS-positive staining, which is indicative of mucoid materials. Other malignant tumors may also originate from the esophagus, including basaloid (B) SCC and carcinoid tumors, and exhibit a polymorphism that is identical to ACC. However, the biological behavior of the two tumors is diverse.

BSCC was initially reported by Wain *et al* in 1986 (20) although the biological characteristics of BSCC were not clearly understood until recently. As a result of this, Tsang *et al* (21) proposed that the numerous ACC cases that have been reported should have been diagnosed as BSCC. The following assumptions are made in order to differentiate between these two carcinomas (22–25): BSCCs are generally composed of dense clusters of small cells with scant cytoplasm. Hyperchromatic nuclei and occasionally polygonal nuclei are observed with pathological mitotic figures that exhibit >10/10 high-power fields. Furthermore, BSCC may occur with various differentiation degrees of SCC, including carcinoma *in situ* and infiltrating carcinoma that present with a solid-lobule pattern, in the center of which may be an acne-like necrosis. The cells around the BSCC usually form nests in a cribriform pattern and deposit basement membrane-like material, which hyalinize between the nests. However, the epithelial surface of the tumor is always malignant. By contrast, squamous cells, central acne-like necrosis and mitotic figures are rarely present in ACC. ACC is predominantly composed of glandular epithelium and myoepithelial cells. IHC may aid with the diagnosis of ACC, particularly when it is difficult to differentiate from BSCC. BSCC only expresses cytokeratin, however, ACC occasionally expresses p63, calponin, smooth muscle actin, S-100 and cluster of differentiation (CD)117, and continuously express cytokeratin (19,25–29). In the present case, the tumor presented with a characteristic adenoid cystic differentiation and positive IHC results of muscle actin and S-100 protein provided additional support to the diagnosis.

The macro-appearance of a submucosa carcinoid of the cardia is usually protruded, which was comparable to what was observed in the present case. Histologically, carcinoids lack the typical cribriform pattern, while observation of a tubule or solid pattern is common, therefore, a definitive differentiation for ACC exhibiting a tubule/solid pattern is required. Thus, IHC is considered to be necessary. Carcinoids often express neurosecretory markers, including neuron specific enolase, CD56, chromogranin A and synaptophysin (30), whereas ACCs do not express such biomarkers.

Radical resection is generally the primary option for treating esophageal ACC. The principles of surgery are the same as those for esophageal carcinoma, including radical resection with free margins and local lymphadenectomy (31). In the present case, the lesion was in the cardia, which infiltrates the stomach body. The patient underwent radical total gastrectomy with D2 lymphadenectomy and Roux-en-Y esophagojejunal anastomosis, and recovered well. Meanwhile, the therapeutic effect of radiotherapy and chemotherapy for esophageal ACC remains controversial. The study by Petursson (5) reported that a combination of chemotherapy with cyclophosphamide, vincristine, adriamycin and cisplatin completely remitted ACC. The TNM staging of the patient in the present study was stage IIB. No chemotherapy was applied and no signs of recurrence or metastasis occurred 30 months following surgery.

The prognosis of esophageal ACC is poor due to early lymphatic and distant metastasis (3,5,30). The average overall survival is only seven months following clinical diagnosis, and nine months subsequent to surgical resection. As was reported, the higher metastatic rate compared with other types of carcinoma was the predominant reason for the relatively poor prognosis of ACC. In the 37 esophageal ACC patients in the study reported by Morisaki *et al* (3), only one patient survived for five years following surgery, 15 patients did not exhibit lymph node metastasis and 11 were alive at the time when the study was reported. The mode of postoperative recurrence was lymph node metastasis in three patients, organ metastasis in four and a combination of the two in five patients. Of the nine patients who exhibited organ metastasis postoperatively, seven were found to have lymph node metastasis during the surgery. These findings indicate that a lack of lymph node metastasis is associated with an improved prognosis for esophageal ACC. Specific relative information regarding nine of the 17 cases that were reported in China is as follows: One patient exhibited anastomotic leakage 10 days following surgery and succumbed to hematemesis on day 22, postoperatively. The remaining eight were alive at the time when the report was published, ~2–46 months following the surgery. In the current case the tumor was relatively large and invaded all layers of the stomach. However, all 23 lymph nodes detected were negative for tumor metastasis. A suspected liver metastatic nodule was identified during surgery; however, the pathological examinations revealed that it was an adenomatous hyperplasia. As a result, the prognosis of this patient may be good. Although the patient has been free from recurrence during the 30 months since surgery, continued regular follow-up is considered to be necessary.

The incidence of adenoid cystic carcinoma derived from the esophagus is relatively low and ACC of the cardia is even rarer. Hence little is known regarding the manefestations and operable treatments for this type of malignancy. The current study presented a case of ACC of the cardia diagnosed by its typical adenoid cystic differentiation and positive IHC results for muscle actin and S-100 protein. BSCC and carcinoids should be carefully differentiated through immunohistological examinations due to the fact that they both exhibit a polymorphism that is identical to ACC. Early lymphatic and distant metastasis are associated with poor survival rates. The primary option for treatment is radical resection, however, the efficacy of radiotherapy and chemotherapy remain uncertain. Case series studies with large samples are necessary in order to acquire more information regarding the clinical manifestations and operable treatments for ACC of the upper digestive tract.

## Figures and Tables

**Table I:** Results of IHC of the resected tumor.

Biomarker	IHC result
Ki-67	10% +
p53	+
C-erbB-2	c
Cytokeratin (AE1/AE3)	+
High-molecular cytokeratin	+
Low-molecular cytokeratin	+
Cytokeratin 5/6 Focal	+
Epithelial membrane antigen	+
Vimentin	+
p63	+
Calponin	+
Smooth muscle actin	+
S-100 Focal	+
CD117	+
CD56	−
CD45/LCA	−
Carcinoembryonic antigen	−
Estrogen receptor	−
Progesterone receptor	−
Chromogranin A	−
Synaptophysin	−

CD, cluster of differentiation; LCA, leukocyte common antigen; IHC, immunohistochemistry.

**Table II:** Clinical data of the present case and 17 other cases of ACC of the esophagus reported in China.

Case (ref)	Age/gender	Location	Biopsy	Macroscopic appearance	Depth of invasion	Metastasis to LN	Distant metastases	Treatment	Outcome
1 (Present)	53/M	Cardia	ACC	Protruding	Serosa	None	None	Surgery	Alive 30 mo.
2 (6)	47/M	Lower	SCC	Protruding	Muscule	None	None	Surgery + chemotherapy (DCVU)	NS
3 (7)	48/M	Middle	Not done	Ulcerative	Submucosa	None	None	Surgery	Alive 17 mo.
4 (8)	59/M	Middle	Focal mild hyperplasia of squamous epithelia	Protruding	Submucosa	None	None	Surgery	Alive 21 mo.
5 (9)	60/M	Lower	SCC	Protruding	Muscule	None	None	Surgery	Succumbed 22 days
6 (10)	64/F	Middle	Leiomyoma	Protruding	Muscule	None	None	Surgery	NS
7–10 (11)	42–62 (Mean, 51)/3M, 1F	2 Middle	1 ACC	Unknown	NS	NS	1 Brain	2 × surgery	NS
		1 Lower	1 SCC					2 ×biopsy	
		1 Upper	1 Negative						
			1 No biopsy						
11 (12)	60/M	Middle	SCC	Protruding	Adventitia	None	NS	Surgery	NS
12 (13)	50/M	Lower	Not done	Ulcerative	Adventitia	None	None	Surgery	Alive 30 mo.
13 (14)	54/F	Middle	Not done	Protruding	Serosa	NS	NS	Surgery	NS
14 (15)	49/M	Middle	ACC	Protruding	Mucosa	None	None	Surgery	Alive 46 mo.
15 (16)	47/M	Lower	Not done	Protruding	Mucosa	None	None	Surgery	Alive 3 mo.
16 (17)	68/F	Lower	Adeno-carcinoma	Ulcerative	NS	NS	NS	Surgery	NS
17 (18)	60/M	Middle	Not done	Unknown	Muscule	Yes	NS	Surgery	Alive 2 mo.
18 (18)	58/M	Middle	Not done	Unknown	Sub-adventitia	Yes	NS	Surgery	Alive 37 mo.

ACC, adenoid cystic carcinoma; DCVU, cisplatin, cyclophosphamide, vomcrostome and uracil; LN, lymph node; M, male; F, female; SCC, squamous cell carcinoma; NS, not stated; mo., month.
